# Avatrombopag as alternative therapy for severe aplastic anemia patients who are intolerant or unresponsive to eltrombopag

**DOI:** 10.3389/fimmu.2024.1393829

**Published:** 2024-07-24

**Authors:** Ting Zhang, Qingling Yu, Xiaoyu Chen, Hui Yang, Yuemin Gong, Yawen Zhang, Xiaoqing Liu, Zhinan Yang, Yu Fang, Xue Yan, Xuan Zhou, Jinning Shi, Guangsheng He

**Affiliations:** ^1^ Department of Hematology, The Affiliated Jiangning Hospital of Nanjing Medical University, Nanjing, China; ^2^ Department of Hematology, Affiliated Jianhu Hospital of Nantong University Xinglin College, Yancheng, China; ^3^ Department of Hematology, The First Affiliated Hospital of Nanjing Medical University, Jiangsu Province Hospital, Collaborative Innovation Center for Cancer Personalized Medicine, Nanjing, China; ^4^ Department of Hematology, Nanjing Hospital Affiliated to Nanjing University of Chinese Medicine, Nanjing Second Hospital, Nanjing, China

**Keywords:** avatrombopag, eltrombopag, severe aplastic anemia, safty, therapy

## Abstract

**Introduction:**

Eltrombopag (EPAG), a thrombopoietin receptor agonist, was approved for the treatment of severe aplastic anemia (SAA) combined with immunosuppressive therapy (IST). However, EPAG contains a typical biphenyl structure, which causes liver function damage.

**Methods:**

Twenty patients with SAA who were intolerant or refractory to EPAG were enrolled in a multicenter prospective registry of the Chinese Eastern Collaboration Group of Anemia (ChiCTR2100045895) from October 2020 to June 2023.

**Results:**

Eight patients who were ineffective to EPAG, six with kidney impairment, and nine with abnormal liver function (two with concomitant liver and kidney impairment) were converted to avatrombopag (AVA) therapy with the median duration of AVA treatment was 6 (3-24) months. 17 cases (85%) achieved trilineage hematological response (HR): complete remission (CR) in 3 cases (15%), good partial remission (GPR) in 4 cases (20%), partial remission (PR) in 10 cases (50%), and no response (NR) in 3 cases (15%). The median time to response was 1.7 (0.5-6.9) months, with 16 cases (94%) achieving response within six months and 17 cases (100%) within 12 months. 9 cases (50%) achieved transfusion independence. AVA converted treatment was associated with higher neutrophil counts (0.8×10^9^/L vs 2.2×10^9^/L, p=0.0003), platelet counts (11×10^9^/L vs 39×10^9^/L, p=0.0008), hemoglobin count (59g/L vs 98g/L, p=0.0002), red cell count (1.06×10^12^/L vs 2.97×10^12^/L, p=0.001), and absolute reticulocyte count (31.99 ×10^9^/L vs 67.05×10^9^/L p=0.0004) were all significantly elevated compared with the pre-treatment level. After the conversion to AVA therapy, liver and kidney function indexes were maintained within the normal range, no AVA related grade 2 or higher adverse events occurred, and no thrombotic events occurred.

**Conclusion:**

The conversion to AVA was an optimal choice for patients with SAA who were EPAG intolerant or refractory.

**Clinical trial registration:**

http://www.chictr.org.cn/showproj.html?proj=125480, identifier ChiCTR2100045895.

## 1 Introduction

Severe aplastic anemia (SAA) is a bone marrow failure syndrome characterized by T lymphocyte-mediated apoptosis of hematopoietic stem cells (1). Intensive immunosuppressive therapy (IST) is the first-line treatment for SAA patients who are not suitable for transplantation, with an effective rate of 60%-70% for anti-thymocyte globulin (ATG) combined with cyclosporine A (CsA) treatment (2).

Interferon-γ can affect the binding of TPO to TPO-R. EPAG, as a small molecule compound, can bypass this action and bind to the transmembrane region of TPO-R, activating the intracellular STAT signaling pathway, increasing the number of CD34^+^ cells and multipotent hematopoietic progenitor cells, and improving the function of hematopoietic stem-progenitor cells (HSPC) (3–5). It can also specifically activate the classical non-homologous end joining DNA repair mechanism, thereby increasing the genetic stability of HSPC (6). EPAG chelates metal cations, which can alleviate iron overload caused by chronic anemia and transfusion dependence, and improve the hematopoietic microenvironment (7). In combination with IST, EPAG could improve therapeutic response (8–10).

However, EPAG contains a typical biphenyl structure, requires the involvement of uridine diphosphoglucuronyl transferase UGT1A1 and UGT1A3 for metabolism. It can cause liver function damage, and the FDA has issued a black box warning for liver toxicity in the EPAG package insert (11).

Unlike EPAG, avatrombopag (AVA) is mainly metabolized by liver enzymes CYP3A and CYP2C9 and does not cause elevation of bilirubin levels (12). AVA does not chelate metal ions and can be taken with food without affecting absorption (13), and absorption is even better when taken with high-fat food (12). AVA has been approved by the FDA for the treatment of thrombocytopenia in patients with chronic liver disease (CLD) during the perioperative period and in adults with chronic immune thrombocytopenia (ITP) (14). The targets and efficacy of different TPO-RA agents are not entirely consistent (15). Therefore, we attempted to switch SAA patients who were intolerant or unresponsive to EPAG to AVA treatment. The cases were prospectively registered in the Severe Aplastic Anemia Database of the Chinese Eastern Collaboration Group of Anemia (CECGA ChiCTR2100045895), and the efficacy and safety of AVA therapy were explored.

## 2 Materials and methods

### 2.1 Study population and methods

#### 2.1.1 Patients

From October 2020 to June 2023, a prospective registration of 20 patients with SAA who switched from EPAG to AVA treatment was conducted and recorded in the CECGA database (ChiCTR2100045895). These patients were from the First Affiliated Jiangning Hospital of Nanjing Medical University, the First Affiliated Hospital of Nanjing Medical University/Jiangsu Province People’s Hospital, and the Second Hospital of Nanjing. All patients signed informed consent forms for treatment and followed ethical principles based on good clinical practice and the Helsinki Declaration. At the same time, we recruited 38 patients who continued to use EPAG as a control (**Supplementary Table 1**).

The diagnostic criteria for SAA were as follows: platelet (PLT) count <20×10^9^/L, absolute neutrophil count (ANC)<0.5 ×10^9^/L, reticulocyte (Ret) count<20 ×10^9^/L. Patients with congenital bone marrow failure disorders, myeloproliferative neoplasms, myelodysplastic syndromes (MDS), classical paroxysmal nocturnal hemoglobinuria (PNH), or acute myeloid leukemia (AML) were excluded from the study (16).

#### 2.1.2 Treatment protocol

Only Rabbit ATG and Porcine ATG were used in our trial, due to the unavailability of horse ATG in China mainland. Porcine ATG at a dose of 30 mg kg^-1^d^-1^ or rabbit ATG at a dose of 3.5 mg kg^-1^d^-1^ were administered continuously for five days. The initial dosage of CsA started at 3-5 mg kg^-1^d^-1^ and was adjusted to maintain the serum trough concentrations at approximately 150-250 μg/L. EPAG was introduced at a dose of 75-100 mg/d, initially from the first day of IST.

For patients who did not respond to IST after six months or those intolerant to EPAG, conversion to AVA at an oral dose of 40 mg/day was initiated. If the platelet count was<50×10^9^/L, the AVA dose was increased to 60 mg/day. If the platelet count was≥150×10^9^/L, the AVA dose was reduced. The dosage range was 20-60 mg/day.

#### 2.1.3 Treatment evaluation criteria response and safety

The treatment response criteria for SAA were based on the revised guidelines of the British Committee for Standards in Haematology in 2016 (16). The treatment response criteria for SAA/very severe aplastic anemia (VSAA) are as follows: (1) Complete Response (CR): Hemoglobin (HGB)>100 g/L, platelet count (PLT)>100×10^9^/L, and absolute neutrophil count (ANC)>1.5×10^9^/L. (2) Good Partial Response (GPR): HGB>80 g/L, PLT>50×10^9^/L, and ANC>1.0×10^9^/L. (3) Partial Response (PR): No longer dependent on blood product transfusions, improvement in hematological parameters, no longer meeting the criteria for SAA, but not reaching the GPR criteria. (4) No Response (NR): Patients who did not achieve independence from blood product transfusion support, or still met the criteria for SAA. Early death within three months after starting AVA therapy was defined as early mortality and considered NR. Overall survival time was defined as the time from the beginning of AVA therapy to patient death or the end of follow-up. Relapse was defined as a decline in blood counts to values requiring re-initiation of full-dose treatment or recurrence of transfusion.

Blood counts and liver and kidney function were examined once a week during the first month, every two weeks within three months, and once a month after three months. Then, the patients were followed up at least every three months after 12 months post-IST.

Kidney function evaluation indexes: all patients underwent urinalysis and serum creatinine at baseline and evaluation, and evidence of proteinuria was considered if urinalysis showed positive urine protein, and glomerular filtration rate [GFR, mL (/min-1.73m^2^)] was estimated according to the MDRD formula (17, 18). According to chronic kidney disease (CKD) stratification (19), CKD is divided into five stages: stage 5 CKD, GFR (/min-1.73m^2^); stage 4 CKD, GFR 15-30mL (/min-1.73m^2^); stage 3 CKD, GFR 30-60mL (/min-1.73m^2^); stage 2 CKD, GFR 60-90mL (/min-1.73m^2^) and evidence of kidney injury; CKD stage 1, GFR >90mL (/min-1.73m^2^) and evidence of kidney injury. Evidence of kidney injury was defined as urinalysis showing proteinuria or abnormal kidney imaging findings (19).

Liver function evaluation indexes: All patients underwent liver function evaluation at baseline and at evaluation, including alanine aminotransferase (ALT), aspartate aminotransferase (AST), glutamyl transpeptide (GGT), alkaline phosphatase (ALP), and hemobilirubin (BIL) respectively. According to the Common Terminology Criteria for Adverse Events version CTCAE 5.0, abnormal liver function is divided into four stages: When the measurement values of ALT and AST are>3.0 times the upper limit, GGT and ALP are>2.5 times the upper limit, BIL is>1.5 times the upper limit, it is defined as CTCAE Grade 1. When the measured values of ALT and AST are 3.0~5.0 times the upper limit, GGT and ALP are 2.5~5.0 times the upper limit, BIL is 1.5~3.0 times the upper limit, it is defined as CTCAE Grade 2. When the measured values of ALT, AST, GGT and ALP are 5.0~20 times the upper limit, BIL is 3.0~10 times the upper limit, it is defined as CTCAE Grade 3. When the measured values of ALT, AST, GGT and ALP are more than 20 times the upper limit, BIL is more than 10 times the upper limit, it is defined as CTCAE Grade 4.

Bone marrow cytology, bone marrow biopsy, and cytogenetic tests were conducted 12 months after AVA therapy to evaluate treatment efficacy and monitor disease progression. Adverse events were graded according to the Common Terminology Criteria for Adverse Events version CTCAE 5.0.

#### 2.1.4 Statistical analysis

Statistical analysis and graph plotting were performed using GraphPad Prism 8.0.2 software. Continuous variables were described as means ± standard deviations. Paired t-tests were used to compare within-group changes before and after treatment. One-way analysis of variance (ANOVA) or Wilcoxon rank-sum test was used to compare changes before and after treatment. Categorical data were described as frequencies (percentages). All statistical tests were two-sided, and the statistical significance was P<0.05.

## 3 Results

### 3.1 Patient characteristics

Among 20 patients with SAA, the median age was 33.5 (3-74) years, with 9 males and 11 females. 9 cases of abnormal liver function (2 cases with concurrent liver and kidney function damage), 6 cases of kidney function damage, 8 cases switched to AVA treatment due to ineffective oral EPAG, including 1 case with liver and kidney function damage, and 1 case ineffective with liver and kidney function damage (**Table 1**).

**Table 1:** Patients clinical features.

Case	Age (years)	Gender	Past Treatment	Duration of EPAG Therapy (months)	Reason for Switching	Therapeutic Effect
1	29	male	ATG+CsA+EPAG	5	Liver damage, Kidney damage	CR
2	49	female	ATG+CsA+EPAG	3	Liver damage	PR
3	10	male	ATG+CsA+EPAG	9	Liver damage	NR
4	24	female	ATG+CsA+EPAG	3	Liver damage	PR
5	16	male	ATG+CsA+EPAG	5	Kidney damage	CR
6	38	female	ATG+CsA+EPAG	5	Invalid	PR
7	74	male	CsA+EPAG	6	Kidney damage	PR
8	66	female	ATG+CsA+EPAG	3	Liver damage,	PR
9	42	female	ATG+CsA+EPAG	14	Invalid,Liver damage,Kidney damage	PR
10	15	male	ATG+CsA+EPAG	9	Kidney damage	GPR
11	11	male	ATG+CsA+EPAG	10	Invalid	PR
12	3	male	ATG+CsA+EPAG	13	Invalid	PR
13	48	female	ATG+CsA+EPAG	6	Liver damage,	PR
14	4	female	ATG+CsA+EPAG	7	Invalid	GPR
15	40	male	ATG+CsA+EPAG	4	Invalid	NR
16	3	female	ATG+CsA+EPAG	4	Invalid	NR
17	22	female	ATG+CsA+EPAG	5	Invalid	GPR
18	16	male	ATG+CsA+EPAG	3	Liver damage,	PR
19	61	male	ATG+CsA+EPAG	5	Kidney damage	GPR
20	41	female	ATG+CsA+EPAG	4	Liver damage,	CR

ATG, Anti-Thymocyte Globulin; CsA, Cyclosporine.

The baseline neutrophil count was 0.81 (0.1-2.99) ×10^9^/L, red blood cell count was 1.06 (0.56-2.73) ×10^12^/L, reticulocyte percentage was 1.7 (0.27-3.12) %, hemoglobin level was 59 (50-115) g/L, and platelet count was 11 (0-58) ×10^9^/L, ARC 31.99 (1.37-60.6) ×10^9^/L.

### 3.2 Efficacy

In total of 20 patients, 17 cases (85%) achieved trilineage hematological response (HR): CR in three cases (15%), GPR in four cases (20%), PR in ten cases (50%), and NR in three cases (15%). Of these, 8 were male and 9 female, with a median age of 39.5 (3-74). The median time to response was 1.7 (0.5-6.9) months, with 16 cases (94%) achieving response within six months and 17 cases (100%) within 12 months. Nine cases (50%) achieved transfusion independence (**Table 1**).

**Figure 1:**
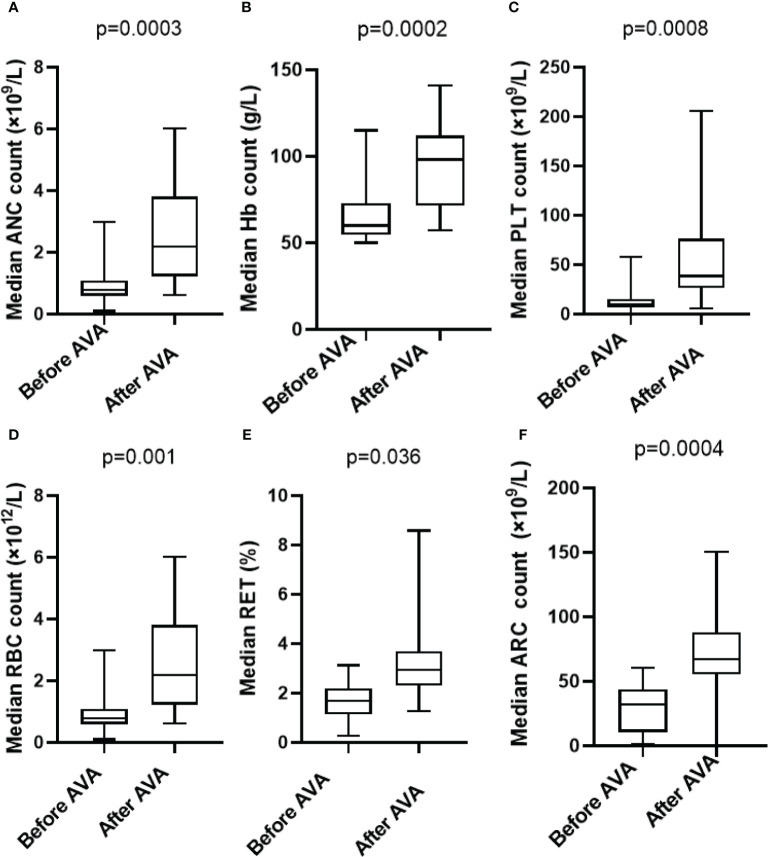
Hematologic improvements in SAA who received AVA: **(A)** Absolute neutrophil count (0.8×10^9^/L vs 2.2 ×10^9^/L,p=0.0003); **(B)** Hemoglobin (59 g/L vs 98 g/L, p=0.0002); **(C)** Blood platelet count (11×10^9^/L vs 39 ×10^9^/L, p=0.0008); **(D)** Red blood cell count (1.06 ×10^12^/L vs 2.97 ×10^12^/L, p=0.001); **(E)** Reticulocyte percentage (1.7% vs 3.4%, p=0.036); **(F)** Absolute reticulocyte count (31.99 ×10^9^/L vs 67.05×10^9^/L p=0.0004).

After the AVA treatment, peripheral blood counts were improved significantly in neutrophil count within 6 months (0.8 ×10^9^/L vs 2.2 ×10^9^/L, p=0.0003), platelet count (11 ×10^9^/L vs 39 ×10^9^/L, p=0.0008), hemoglobin level (59g/L vs 98g/L, p=0.0002), red blood cell count (1.06 ×10^12^/L vs 2.97 ×10^12^/L, p=0.001), and reticulocyte percentage (1.7% vs 3.4%, p=0.036), absolute reticulocyte count (31.99 ×10^9^/L vs 67.05×10^9^/L p=0.0004) (**Figure 1**).

Univariate and multivariate analyses revealed that age, gender, medication duration, ANC, Hb, PLT, and RET showed no significant influences on the response in patients using AVA, while ARC significantly influenced the response in patients using AVA (**Table 2**).

**Table 2:** Factors related to the efficacy response of AVA therapy with univariate and multivariate analysis.

Category and variable	P value	
	Univariate	Multivariate
Age	0.273	0.330
Gender	0.556	0.825
Medication duration	0.788	0.576
ANC (×109/L)	0.258	0.199
Hb (g/L)	0.973	0.820
PLT (×109/L)	0.847	0.897
RET (%)	0.181	0.125
ARC (×109/L)	0.023	0.029

ANC, absolute neutrophil count; Hb, Hemoglobin count; PLT, blood platelet count; RET Reticulocyte percentage; ARC, absolute reticulocyte count.

In the 8 cases of ineffective EPAG, 6 cases (75%) achieved hematological response (HR) after switching to AVA treatment, including 2 cases (25%) with good partial response (GPR) and 4 cases (50%) with partial response (PR).

There was no statistically significant difference in overall efficacy between patients conversion to AVA and 38 patients who continued to use EPAG over the same period (85% vs 87%, p=0.103) (**Supplementary Table 2**), suggesting that the efficacy of patients who switched to AVA due to inefficacy or liver or kidney impairment was comparable to that of patients who continued to use EPAG (**Table 3**).

**Table 3:** Comparison between patients that switched to Avatrombopag and patients that continued Eltrombopag treatment.

	AVA group (n=20)	EGAP group (n=38)	*P*
Age (years, median, range)	33.5 (3~74)	36.2 (18-74)	0.928
Gender (male/female)	9/11	17/22	0.658
Severity of aplastic anemia, n (%)			
Severe Very severe	19 1	29 9	0.097
Percentage of patients			
HR	17 (85%)	33 (87%)	0.103
Median follow-up time	34.5 (18-58)	39 (15-65)	0.956

AVA, Avatrombopag; EGAP, Eltrombopag; HR, hematological response.

### 3.3 Safety

**Figure 2:**
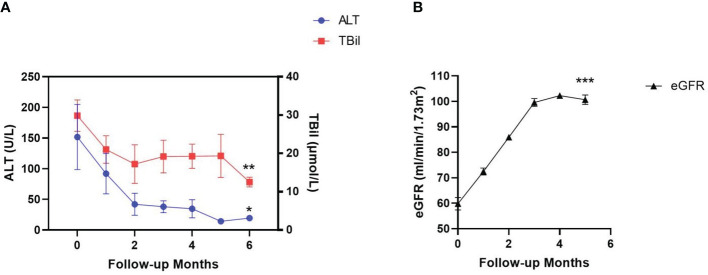
Changes of liver and kidney function indicators during AVA treatment. Liver function was evaluated by alanine aminotransferase (ALT) and total bilirubin (TBil) level in the patients’ serum **(A)**. kidney function was evaluated by estimated glomerular filtration rate (eGFR) **(B)**. Data are shown as Mean ± SEM. *p<0.05; **p<0.01; ***p<0.001.

The median duration of AVA treatment was 6 (3-24) months. In the 9 patients who were intolerant to previous EPAG treatment due to liver dysfunction, the liver function did not worsen after switching to AVA treatment and showed significant improvement in the 6th month after treatment (**Figure 2A**). ALT (151 VS 21) U/L (P=0.004), TBIL (30 VS 12) μmol/L (P=0.0003), DBIL (15 VS 5) μmol/L (P=0.003), and IBIL (14 VS 7) μmol/L (P=0.004) (**Table 4**). 8 cases achieved hematological response (HR) (89%), including 2 with complete response (CR) (22%) and 6 with partial response (PR) (67%). Among the 6 patients with kidney damage, 3 had stage 3 eGFR impairment and 3 had stage 2 eGFR impairment. After AVA therapy, all eGFR levels improved to grade 1 without new adverse events (**Figure 2B**). EGFR (58.7 VS 100.7) mL (/min-1.73m^2^) (P<0.0001) (**Table 4**). All patients (100%) achieved HR, including two with CR (33%), two with GPR (33%), and two with PR (33%).

**Table 4:** Changes in liver and kidney function indicators before and after treatment with AVA.

Time points	ALT (U/L)	AST (U/L)	ALP (μmol/L)	GGT (μmol/L)	TBIL (μmol/L)	DBIL (μmol/L)	IBIL (μmol/L)	eGFR
**Before AVA**	151 (19 -367)	146 (18-420)	106 (44-186)	73 (13-239)	30 (8-41)	15 (4-29)	14 (4-24)	58.7 (53.9-68)
**After AVA**	21 (11-41)	18 (12-25)	69 (46-86)	21 (9-35)	12 (7-16)	5 (2-8)	7 (5-8)	100.7 (95-105.8)
***T or Z* value**	2.25	1.95	1.6	1.62	3.8	2.5	2.3	13.2
***P* value**	0.004	0.08	0.14	0.13	0.003	0.03	0.04	<0.0001

ALT, Alanine aminotransferase; AST, Aspartate aminotransferase; ALP, Alkaline phosphatase; GGT, Gamma-glutamyltransferase; TBIL, Total bilirubin; DBIL, Direct bilirubin; IBIL, Indirect bilirubin.

The duration of the median follow-up was 34.5 (18-58) months. During the AVA treatment, there were no patient death and no grade 2 or higher adverse events were observed. No patients developed bone marrow fibrosis, myelodysplastic syndrome (MDS), acute myeloid leukemia (AML), or paroxysmal nocturnal hemoglobinuria (PNH) clone transformation. In addition, the liver and kidney functions remained within normal ranges with no occurrence of thrombotic events.

## 4 Discussion

The prospective cohort study (8) and the randomized controlled study (4) on the combination of IST and EPAG as first-line treatment for SAA have shown that EPAG can accelerate hematologic response, effectively improving the quality of hematologic response and increasing the overall response rate in patients. EPAG can inhibit UDP-glucuronosyltransferase 1A1 (UGT1A1) and Organic Anion Transporting Polypeptide 1B1 (OATP1B1), resulting in elevated levels of indirect bilirubin. Common adverse reactions include a mild to moderate increase in serum indirect bilirubin without the need for dosage adjustment, an 18% incidence of grade 3-4 ALT elevation, an 18% incidence of TBIL elevation, and a 12% incidence of AST elevation. Some patients may require dose reduction or temporary discontinuation of EPAG (20). In Phase II clinical trials for the treatment of refractory SAA, some patients also exhibited significant elevation in transaminase levels.

Liver decompensation, mainly ascites and liver encephalopathy, was seen at higher rates in the EPAG group compared with the placebo group (10 vs 5%) (21). Another randomized, placebo-controlled trial with planned enrollment of 500 patients was terminated early due to an increased incidence of thrombotic events among EPAG treated patients (22).

In two placebo-controlled Phase III studies conducted in pediatric chronic ITP patients, 4.7% of patients in the EPAG group and 0% of patients in the placebo group reported ALT levels ≥3 times the upper limit of normal (ULN) as adverse events (11).

Following a single oral dose of 50mg EPAG, the area under the plasma concentration-time curve (AUC inf) of EPAG decreased by 32% in patients with mild kidney impairment, 36% in patients with moderate kidney impairment, and 60% in patients with severe kidney impairment. Due to a decreasing trend shown by the exposure to EPAG in patients with kidney impairment, a close monitoring of kidney function, such as measuring serum creatinine and/or urine analysis, is required for patients with kidney impairment (11). EPAG should be used with caution in patients with kidney impairment.

SAA patients often require long-term use of CsA, which has toxic effects on the kidneys and liver. CsA causes kidney vasoconstriction, increased vascular resistance, decreased kidney blood flow, and reduced glomerular filtration (23). The liver is the primary site of CsA metabolism, hence when liver function is abnormal, particularly in cases of moderate to severe impairment, the activity of CYP3A (Cytochrome P450IIIA enzymes) and bile secretion are affected, thus impacting the absorption and metabolism of CsA. Klintmalm et al. (24) reported CsA-induced hepatotoxicity, with 13 out of 66 patients (19.7%) experiencing liver function abnormalities and at least one episode of hepatotoxicity, which is defined as a bilirubin level exceeding 2.0mg/100ml (34.2mmol/L) or unconjugated bilirubin accounting for 50% of the elevation. Half of the 13 patients had levels of AST and ALT three times higher than normal values.

AVA is metabolized in the liver via cytochrome P450 enzymes (CYP2C9 and CYP3A4), with 88% undergoing intestinal metabolism (with 34% remaining unmetabolized) and 6% undergoing kidney metabolism (12). Age (18-86 years), body weight, gender, race, and any degree of liver damage or mild to moderate kidney impairment (creatinine clearance ≥30 mL/min) do not affect the pharmacodynamics of AVA, and AVA can be used in patients with kidney insufficiency. Two phase III studies (ADAPT-1 and ADAPT-2) evaluated the safety and efficacy of AVA in increasing platelet counts in patients with chronic liver disease (CLD) and thrombocytopenia (25). Regardless of whether patients received a daily dose of 60mg AVA or 40mg AVA, most adverse events (AEs) were mild to moderate in severity. The most commonly observed treatment-emergent adverse events (TEAEs), including abdominal pain, diarrhea, dyspepsia, nausea, fever, dizziness, and headache, were similar between the AVA and placebo groups, and no liver or kidney function damage caused by AVA was observed.

Considering the efficacy of AVA in chronic ITP and its lack of significant liver and kidney toxicity, we attempted to use AVA in SAA patients who were intolerant to EPAG treatment. In this study, nine SAA patients with liver function abnormalities showed gradual normalization of liver function after switching to AVA treatment. Six patients with kidney impairment achieved improved estimated glomerular filtration rate (eGFR) after AVA treatment, without any adverse events. In this study, at the last follow-up, the longest user had been using AVA for 24 months, and no abnormalities were detected in liver and kidney function indicators.

The pharmacokinetics of EPAG exhibit dose-dependency and linearity, with EPAG increasing platelet count in a dose-dependent manner (26, 27). The hematologic response in patients with aplastic anemia (AA) is also dose-related (28). However, EPAG often induces hepatotoxicity at higher doses, requiring co-administration with CsA, which has hepatokidney toxicity, in the treatment of SAA. This limitation restricts the full dosage and duration of EPAG therapy (29, 30). *In vitro* studies have shown that TPO-RAs stimulate proliferation of Ba/F3 cells expressing human c-MPL in a concentration-dependent manner, promoting differentiation of human cord blood CD34^+^ cells into megakaryocytes. The 50% effective concentration (EC50) of AVA is 3.65-6.84 times higher than EPAG, indicating that AVA is significantly more potent (15). AVA has minimal impact on liver and kidney function, allowing for adequate dosing, which may lead to better clinical efficacy. Ultimately, 70% of patients who were unresponsive to EPAG showed neutrophil, red blood cell, or platelet responses to AVA, suggesting differences in drug metabolism and efficacy between different molecular structures of EPAG and AVA. However, whether cross-tolerance exists between these drugs remains to be elucidated.

While EGAP treatment for AA may carry the risk of clonal evolution and bone marrow fibrosis (31), it remains uncertain whether AVA, which acts through similar mechanisms, poses the same risks. In this study, all 20 patients had normal karyotype before treatment, and no clonal evolution or bone marrow fibrosis has been observed thus far. However, the duration of the follow-up period remains limited, and further observation is required.

Zaimoku Y and Li R et al. indicated that the addition of EPAG to IST markedly improved responses in patients who had a higher ARC (32, 33). ARC could help clinically assess bone marrow function since the robust recovery of ARC following IST could predict long-term survival (33). In our study, ARC also showed significance in both univariate and multivariate analyses, and a subsequent increase in sample size is needed to verify its relationship with the efficacy of AVA treatment.

Preliminary results suggest that AVA has some efficacy in patients with SAA who are difficult to treat or intolerant to standard IST combined with EPAG and good tolerability. However, both the sample size and the follow-up period of the study are limited, necessitating long-term follow-up to evaluate long-term efficacy, risk of clonal evolution, and other factors.

## Data availability statement

The original contributions presented in the study are included in the article/**Supplementary Material**. Further inquiries can be directed to the corresponding author.

## Ethics statement

The studies involving humans were approved by the First Affiliated Hospital of Nanjing Medical University Ethic Committee. The studies were conducted in accordance with the local legislation and institutional requirements. Written informed consent for participation in this study was provided by the participants’ legal guardians/next of kin.

## Author contributions

TZ: Validation, Supervision, Software, Project administration, Methodology, Investigation, Formal analysis, Data curation, Conceptualization, Writing – review & editing, Writing – original draft. QY: Validation, Supervision, Software, Project administration, Methodology, Investigation, Formal analysis, Data curation, Conceptualization, Writing – review & editing, Writing – original draft. XC: Writing – original draft, Validation, Data curation. HY: Writing – original draft, Investigation, Data curation. YG: Writing – review & editing, Project administration, Methodology, Formal analysis. YZ: Writing – review & editing, Software, Conceptualization. XL: Writing – original draft, Methodology, Data curation. ZY: Writing – original draft, Investigation, Data curation. YF: Writing – original draft, Methodology, Data curation. XY: Writing – original draft, Project administration, Methodology. JS: Writing – review & editing, Supervision, Formal analysis. XZ: Writing – original draft, Data curation. GH: Visualization, Validation, Supervision, Resources, Project administration, Methodology, Funding acquisition, Formal analysis, Data curation, Writing – review & editing, Writing – original draft.
