# Supplementary material for: Avatrombopag as alternative therapy for severe aplastic anemia patients who are intolerant or unresponsive to eltrombopag
Source: Front Immunol. 2024 Jul 24;15:1393829. doi: 10.3389/fimmu.2024.1393829 (PMC11303196; doi:10.3389/fimmu.2024.1393829)
Supplement: Supplementary file 1 [file Table_1.docx]

Supplementary Table 1 38 patients who continued to use EPAG specific information

| Case | Age (years) | Gender | degree of seriousness | Duration of EPAG Therapy (months) | Therapeutic Effect |
| --- | --- | --- | --- | --- | --- |
| 1 | 73.9 | female | NSAA | 45 | NR |
| 2 | 20.3 | female | SAA | 15 | NR |
| 3 | 27.0 | female | SAA | 15 | CR |
| 4 | 49.5 | female | NSAA | 31 | CR |
| 5 | 58.5 | female | SAA | 33 | GPR |
| 6 | 65.1 | male | SAA | 53 | GPR |
| 7 | 51.2 | male | SAA | 39 | NR |
| 8 | 20.9 | male | SAA | 36 | PR |
| 9 | 37.6 | male | SAA | 34 | CR |
| 10 | 62.7 | male | SAA | 36 | CR |
| 11 | 48.0 | female | NSAA | 42 | NR |
| 12 | 34.8 | male | NSAA | 40 | CR |
| 13 | 31.4 | male | SAA | 36 | CR |
| 14 | 67.8 | female | SAA | 39 | CR |
| 15 | 44.6 | female | SAA | 35 | CR |
| 16 | 67.6 | female | SAA | 36 | GPR |
| 17 | 34.4 | male | SAA | 32 | PR |
| 18 | 28.1 | female | NSAA | 33 | CR |
| 19 | 22.5 | male | NSAA | 32 | PR |
| 20 | 52.8 | female | SAA | 34 | CR |
| 21 | 27.1 | female | SAA | 33 | CR |
| 22 | 66.3 | male | SAA | 48 | PR |
| 23 | 38.7 | female | SAA | 52 | CR |
| 24 | 48.0 | female | NSAA | 31 | CR |
| 25 | 33.1 | female | SAA | 32 | GPR |
| 26 | 35.7 | male | SAA | 32 | NR |
| 27 | 62.5 | female | SAA | 34 | PR |
| 28 | 26.7 | male | NSAA | 33 | CR |
| 29 | 19.2 | female | SAA | 36 | PR |
| 30 | 38.7 | female | SAA | 37 | CR |
| 31 | 28.5 | male | SAA | 39 | PR |
| 32 | 62.8 | male | SAA | 38 | PR |
| 33 | 29.7 | female | SAA | 40 | CR |
| 34 | 21.5 | male | SAA | 39 | CR |
| 35 | 56.2 | female | SAA | 41 | CR |
| 36 | 18.3 | male | SAA | 56 | PR |
| 37 | 70.8 | female | NSAA | 41 | PR |
| 38 | 19.3 | male | SAA | 65 | PR |
