# Supplementary material for: Avatrombopag as alternative therapy for severe aplastic anemia patients who are intolerant or unresponsive to eltrombopag
Source: Front Immunol. 2024 Jul 24;15:1393829. doi: 10.3389/fimmu.2024.1393829 (PMC11303196; doi:10.3389/fimmu.2024.1393829)
Supplement: Supplementary file 2 [file Table_2.docx]

Supplementary Table 2 Compared effective with AVA and EPAG

|  | effective | ineffective | P value |
| --- | --- | --- | --- |
| AVA | 17 | 3 | 0.103 |
| EPAG | 33 | 5 |  |
